# Multimodal imaging and genetic characteristics of Chinese patients with *USH2A*‐associated nonsyndromic retinitis pigmentosa

**DOI:** 10.1002/mgg3.1479

**Published:** 2020-09-06

**Authors:** Chong Chen, Qiao Sun, Mingmin Gu, Tianwei Qian, Dawei Luo, Kun Liu, Xun Xu, Suqin Yu

**Affiliations:** ^1^ Department of Ophthalmology Shanghai General Hospital Shanghai Jiao Tong University Shanghai China; ^2^ National Clinical Research Center for Eye Diseases Shanghai Key Laboratory of Ocular Fundus Diseases Shanghai Engineering Center for Visual Science and Photomedicine Shanghai Engineering Center for Precise Diagnosis and Treatment of Eye Diseases Shanghai China; ^3^ Department of Medical Genetics School of Medicine Shanghai Jiao Tong University Shanghai China

## Abstract

**Background:**

To determine the clinical characteristics and molecular genetic background responsible for *USH2A* mutations associated with nonsyndromic retinitis pigmentosa (RP) in five Chinese families, a retrospective cross‐sectional study was performed.

**Methods:**

Data on detailed history and comprehensive ophthalmological examinations were extracted from medical charts. Genomic DNA was sequenced by whole‐exome sequencing. The pathogenicity predictions were evaluated by *in silico* analysis. The structural modeling of the wide‐type and mutant USH2A proteins was displayed based on the I‐Tasser software.

**Results:**

The ultra‐wide‐field fundus imaging showed a distinctive pattern of hyperautofluorescence in the parafoveal ring with macular sparing. Ten *USH2A* variants were detected, including seven missense mutations, two splicing mutations, and one insertion mutation. Six of these variants have already been reported, and the remaining four were novel. Of the de novo mutations, the p.C931Y and p.G4489S mutations were predicted to be deleterious or probably damaging; the p.M4853V mutation was predicted to be neutral or benign; and the IVS22+3A>G mutation was a splicing mutation that could influence mRNA splicing and affect the formation of the hairpin structure of the USH2A protein.

**Conclusions:**

Our data further confirm that USH2A protein plays a pivotal role in the maintenance of photoreceptors and expand the spectrum of *USH2A* mutations that are associated with nonsyndromic RP in Chinese patients.

## Graphical abstract

This study identified novel mutations of the USH2A gene using a whole‐exome sequencing approach in nonsyndromic RP patients. RP patients with USH2A mutations have a distinctive pattern in ultra‐wide‐field fundus autofluorescence.

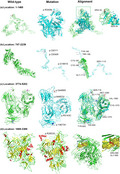

## 1 INTRODUCTION

Retinitis pigmentosa (RP) is the most common inherited retinal degeneration and occurs in approximately 1 in 4000 individuals worldwide (den Hollander, Black, Bennett, & Cremers, 2010). Typical RP is heralded by primary rod degeneration leading to night blindness and the development of tunnel vision, while the loss of cone function leads to the deterioration of central vision (Ayuso & Millan, 2010; Pierrache et al., 2016). The typical clinical signs of RP include retinal arteriolar attenuation and a generalized and diffuse pattern of mottled and moth‐eaten retinal pigment epithelium (RPE). However, the disease onset, progression, retinal characteristics, and visual prognosis may vary remarkably among patients, even within the same family (Freund, Sarraf, Mieler, & Yannuzzi, 2017; Hartong, Berson, & Dryja, 2006). In addition, its early symptoms and signs can be atypical, which may result in misdiagnosis.

RP has various inheritance patterns, including an autosomal dominant (AD) pattern (30–40%), an X‐linked pattern (10–15%), and an autosomal recessive (AR) pattern (50–60%) (Freund et al., 2017). Sporadic cases account for approximately 40% of all cases, although these data vary between different populations (Neveling, Collin, & Gilissen, 2012).

To date, nearly 3100 pathogenic mutations have been reported in more than 80 genes associated with nonsyndromic RP, and 55 of these mutations have been related to arRP (Daiger, Sullivan, & Bowne, 2013; Huang et al., 2015; Perez‐Carro et al., 2016). Although the functions of some of these genes have been studied extensively, many of them confer different phenotypes. It is, therefore, difficult to establish a clear genotype‐phenotype correlation, since different genes cause distinct or partially overlapping clinical phenotypes (Berger, Kloeckener‐Gruissem, & Neidhardt, 2010).

Mutations in *Usher syndrome 2A* (*USH2A*; OMIM 608400) are a considerable cause of RP and can result in two distinct phenotypes: nonsyndromic RP and Usher syndrome type IIa. Patients with Usher syndrome type IIa experience both RP and congenital sensorineural hearing impairment, while patients with nonsyndromic RP are without extraocular symptoms (Pierrache et al., 2016).

In this study, we applied a whole‐exome sequencing approach and copy number variation (CNV) analysis to test RP‐related mutations in 25 patients clinically diagnosed with RP. Of them, five unrelated patients (probands) were identified with ten *USH2A* mutations, including four novel heterozygous mutations in *USH2A*. With *in silico* and functional prediction, we further explored the potential pathogenic effects of these mutations, which may represent possible mechanisms for nonsyndromic RP. This study is the first to model the three‐dimensional structures of wild‐type and mutant USH2A proteins, which is more intuitive to understand the possible effects of protein structural changes in protein functions.

In addition, we evaluated the retinal imaging features of patients with nonsyndromic RP resulting from *USH2A* mutations. With multimodal imaging, different stages of the disease among patients were compared, which may provide new insights into the natural progress of *USH2A*‐associated nonsyndromic RP.

## 2 METHODS

### 2.1 Ethical compliance

This study was approved by the medical ethics committee of Shanghai General Hospital, Shanghai Jiao Tong University (No. 2019KY044), and conducted in accordance with the Declaration of Helsinki.

### 2.2 Study population and clinical examination

Patients assessed at Shanghai General Hospital with an established diagnosis of RP were included. Except for visual problems, these patients did not have other health‐related issues.

All patients underwent a comprehensive ophthalmologic evaluation including BCVA, a slit‐lamp examination, ultra‐wide‐field color fundus photography (Heidelberg Engineering, Heidelberg, Germany), ultra‐wide‐field FAF (Heidelberg) imaging, a spectral‐domain (SD) OCT scan (Heidelberg), and an ERG (Electrophysiological Diagnostic Unit Retimax, Roland Consult, Brandenburg, Germany). Ultra‐wide‐field fluorescein angiography (FFA; Heidelberg) was performed on all participants who agreed to collaborate. The diagnosis of RP was based on clinical manifestations, typical biomicroscopic findings in the fundus, FAF, FFA, OCT, and ERG. Atypical presentations of RP were also included. After enrollment, the diagnosis was further confirmed by genetic testing. We included siblings, parents, and offspring of the probands in our cohort. ERG was not a focus of this study, but five probands did undergo ERGs performed according to an International Society for Clinical Electrophysiology of Vision (ISCEV) protocol (McCulloch et al., 2015; McGuigan et al., 2017).

### 2.3 Genetic analysis

Genomic DNA was extracted from surplus peripheral blood leukocytes isolated from previous samples collected from diagnosed individuals using QIAamp DNA Blood Midi Kits (Qiagen, Hilden, Germany) according to the manufacturer's protocols.

To obtain a molecular diagnosis, all probands were submitted to whole‐exome sequencing using an xGen Exome Research Panel v1.0 (Integrated DNA Technologies, USA) on an Illumina NovaSeq 6000 platform (Illumina, USA). The xGen Exome Research Panel v1.0 consists of 429,826 individually synthesized and quality‐controlled xGen Lockdown® Probes. The Exome Research Panel spans a 39‐Mb target region (19,396 genes) of the human genome and covers 51 Mb of end‐to‐end tiled probe space. All probes in the panel are manufactured according to GMP standards. Mass spectrometry and OD measurements are taken for each probe to ensure appropriate representation of the correctly manufactured probes in the pool (https://sg.idtdna.com/pages/​produ​cts/next-gener​ation​-seque​ncing/​hybri​dizat​ion-captu​re/lockd​own-panel​s/xgen-exome​-resea​rch-panel). More than 95% of the targeted sequences were covered plenarily for high‐confidence variant calling (>20 X coverage; mean coverage depth of over 100X). The paired‐end alignment was performed using BWA aln to the 1000 genomes hg19/GRCh37 reference genome. SAM files were sorted and converted to BAM, and duplicates were marked with Picard. GATK was applied for local realignment and base quality score recalibration, and variants were called jointly in all samples using the GATK's HaplotypeCaller in the “GENOTYPE_GIVEN_ALLELES” mode (Wang et al., 2018). CNV mutation frequency information was obtained by searching the Exome Aggregation Consortium (ExAC) website (http://exac.broad​insti​tute.org/), which includes data obtained from 60706 unrelated individuals sequenced as part of various disease‐specific and population genetic studies. Through ExAC, the allele frequencies of the variants can be determined and filtered by a 0.01 standard.

Sanger sequencing was subsequently applied to further validate the identified mutation(s). Linkage analysis was performed by direct sequencing among the available family members.

### 2.4 Bioinformatics analyses

Conservative Prediction: Information about the structure of the *USH2A* gene (NG_009497.1) was acquired by searching the Ensembl database (http://asia.ensem​bl.org), and a phylogenetic tree of the gene was drawn using the Genetree tool. The sequence of multiple alignments of the USH2A homologous family was extracted from the phylogenetic tree data, and the evolutionary conservativeness of amino acids corresponding to each mutation was calculated. The seqlogo map of the amino acid sequence around the mutation site was drawn by WebLogo (http://weblo​go.berke​ley.edu/), and the conservativeness of the amino acid was determined from the map (Crooks, Hon, Chandonia, & Brenner, 2004).

Pathogenicity Prediction: The sequence of protein O75445, which corresponds to the *USH2A* gene, was obtained from the UniProt database (http://www.unipr​ot.org/). The protein sequence was used as the input sequence, and the amino acid location of the mutation was selected. The pathogenicity of the mutation site was predicted using the PolyPhen‐2 database (http://genet​ics.bwh.harva​rd.edu/pph2/) (Adzhubei et al., 2010).

Additionally, the chemical dissimilarity of codon replacements was predicted by Grantham scores, which are categorized into four classes: conservative (0–50), moderately conservative (51–100), moderately radical (101–150), or radical (≥151) according to the classification proposed by Li et al. (Grantham, 1974; Li, Wu, & Luo, 1984). Moreover, the possible functional impact of an amino acid change was predicted by PROVEAN (http://prove​an.jcvi.org/genome_submit_2.php) and SIFT (http://sift.jcvi.org/).

Prediction and analysis of the secondary structure of DNA around the intron mutation: The secondary structure prediction software Mfold (http://unafo​ld.rna.albany.edu/) was used to predict the secondary structure of DNA around the intron splice site mutation IVS22+3A>G (rs117798425). The left 200 bp‐long and right 200 bp‐long sequences of the mutation point was selected as the input sequence, and the folding temperature parameter was set to 37 degrees for the calculation (Zuker, 2003).

Protein structure modeling and analysis: The USH2A protein sequence was retrieved from the UniProt database, and the domain of the sequence was cut by the principle of covering the mutation point to the greatest extent. I‐Tasser software (https://zhang​lab.ccmb.med.umich.edu/I-TASSE​R/) was used to simulate the protein structure before and after mutation. The wide‐type and mutant protein structure were then analyzed using TM‐align (https://zhang​lab.ccmb.med.umich.edu/TM-align/) (Roy, Kucukural, & Zhang, 2010; Yang & Zhang, 2015; Zhang, Freddolino, & Zhang, 2017).

Mutation Point Protein Function Prediction: The functional characteristics of amino acids in the USH2A protein were searched using the UniProt database. The disulfide bonds of USH2A were predicted by DISULFIND software (http://disul​find.dsi.unifi.it/) (Ceroni, Passerini, Vullo, & Frasconi, 2006).

## 3 RESULTS

### 3.1 Clinical features

**FIGURE 1:**
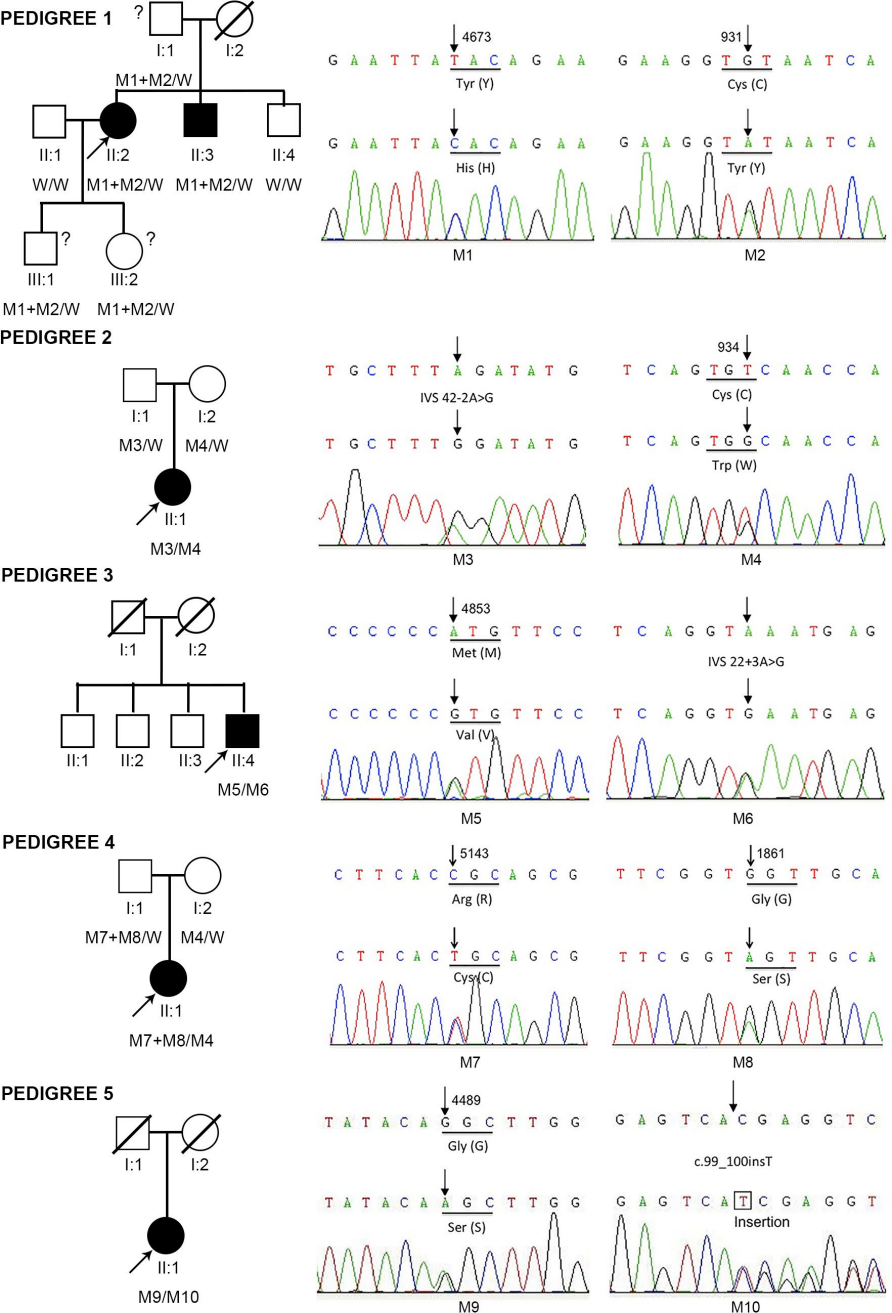
The five Chinese pedigrees with nonsyndromic retinitis pigmentosa and mutations in the *USH2A* gene. Squares and circles indicate males and females, respectively; Arrows symbolize the probands; Black and white denote the status of family members affected or unaffected, respectively; Question marks next to the square or circle indicates the questionable status of the family members. The genotype of each evaluated individual is listed below the individual's symbol and identification number. W: wild type; M1: p.C931Y; M2: p.Y4673H; M3: p.R2853G (IVS42‐2A>G); M4: p.934 W; M5: p.M4853V; M6: IVS22+3A>G; M7: p.R5143C; M8: p.G1861S; M9: p.G4489S; M10: R34Sfs

Among 25 patients clinically diagnosed with RP, 10 eyes of five probands from five pedigrees with nonsyndromic RP resulting from *USH2A* mutations were included (Figure 1). There were two males and three females in the identified probands. The age at presentation ranged from 31 to 60 years, the age of onset ranged from 12 to 46 years. The median follow‐up was 46 months (range: 40–50 months). In the better eye, two patients (40%) had normal visual acuity or mild visual loss (0.9–0.6), one patient (20%) showed moderate visual impairment (0.5–0.2), and the other two patients (40%) presented low vision (<0.2). The patients' demographic features are summarized in Table 1.

**TABLE 1:** Clinical characteristics of probands with nonsyndromic retinitis pigmentosa

Pedigree no.	Proband no.	Gender	Age	Onset	Inheritance	BCVA OD/OS	Follow‐up (months)
1	1‐II:2	Female	47	46	Unknown	0.25/0.3	50
2	2‐II:4	Female	37	37	AR	1.0/0.9	46
3	3‐II:1	Male	60	45	AR	0.03/0.05	44
4	4‐II:1	Male	31	31	AR	1.0/0.9	46
5	5‐II:1	Female	54	12	AR	0.03/0.01	40

Abbreviations: AR, autosomal recessive; OD, oculus dexter; OS, oculus sinister.

Fifteen family members from five pedigrees participated in our study (including five probands), and all of them were tested for whole‐exome sequencing or verified by Sanger sequencing. All participants denied hearing impairment or deafness or balance problems. Anterior segment examinations were unremarkable. The clinical and genetic details of each pedigree were described in the supplemental materials.

### 3.2 Multimodal imaging and disease progression

**FIGURE 2:**
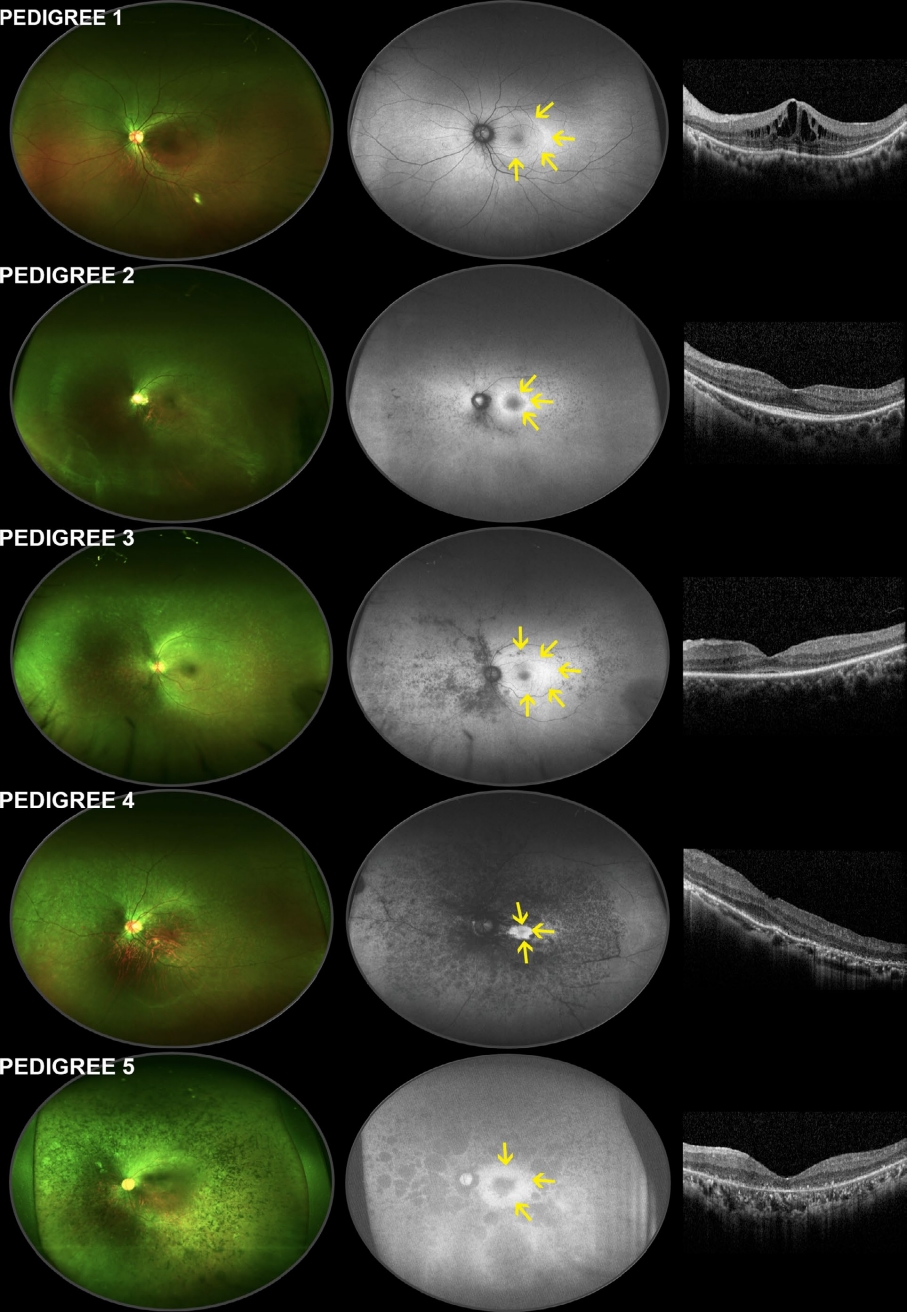
Multimodal images of the probands with *USH2A* mutations in five pedigrees. Ultra‐wide‐field fundus photographs are shown to the left, ultra‐wide‐field fundus autofluorescence images in the middle and optical coherence tomographic images to the right. Only left eyes are shown, but findings in five probands presented a high degree of bilateral symmetry. The yellow arrows indicate the margin of the hyperautofluorescence in the parafoveal ring

In the early stage of RP, the pigment deposits of the RPE are punctate or bone spicule‐shaped (Figure 2, pedigree 1) in the ultra‐wide‐field fundus autofluorescence (FAF) and progressively aggravated into a mottled appearance in the mid periphery of the retina (Figure 2, pedigree 2) with the course advancing. In the advanced stage, a lobular RPE defect along retinal vessels could be seen in the mid periphery of retina (Figure 2, pedigrees 3 and 4). In the late stage, patchy RPE defects confluent into a zonal area of atrophy in the mid and far periphery of the retina with macular sparing (Figure 2, pedigree 5). Macular SD‐OCT showed perifoveal outer retinal atrophy as well as intraretinal cysts in all the probands (Figure 2). More information about the images was described in supplemental materials.

Moreover, although the multimodal fundus appearances varied among different patients, they shared something in common. All the probands showed the distinctive pattern of diffuse and homogeneous hyperautofluorescence parafoveal ring with macular sparing, which could be observed in ultra‐wide‐field FAF (Oh et al., 2019).

### 3.3 Genetic and bioinformatics analyses

In total, 17 sequence variants (including ten *USH2A* variants) were identified in five probands. Among the ten variants of the *USH2A* gene, seven were missense mutations, two were splicing mutations, and one was an insertion mutation. The c.2802T>G (p.934C>W) variant was recurrent and was found in 2/5 probands (40%), 2/10 *USH2A* variants (20%) in our study, but almost all of the patients carried private sequence variants. Six of the variants have already been reported in the literature (Dai, Zhang, & Zhao, 2008; Huang et al., 2013; Jiang et al., 2015; Miyagawa, Naito, Nishio, Kamatani, & Usami, 2013; Xu et al., 2011), while the remaining four were novel (Table 2).

**TABLE 2:** Identified mutations in *USH2A* in patients with nonsyndromic retinitis pigmentosa

Pedigree no.	Mutation no.	Gene	Exon	Type	cDNA	Amino acid	Genotype	Reported
1	M1	*USH2A*	13	Missense	c.2792G>A	C931Y	Hetero	Novel
	M2	*USH2A*	64	Missense	c.14017T>C	Y4673H	Hetero	Reported (McGuigan et al., 2017)
2	M3	*USH2A*	42	Splicing	IVS42‐2A>G	R2853G	Hetero	Reported (McCulloch et al., 2015)
	M4	*USH2A*	13	Missense	c.2802T>G	C934W	Hetero	Reported (Miyagawa et al. 2013; Neveling et al., 2012)
3	M5	*USH2A*	66	Missense	c.14557A>G	M4853V	Hetero	Novel
	M6	*USH2A*	Intro n	Splici ng	IVS22+3A>G	Splicing	Hetero	Novel
4	M7	*USH2A*	71	Missense	c.15427C>T	R5143C	Hetero	Reported (Oh et al., 2019)
	M8	*USH2A*	28	Missense	c.5581G >A	G1861S	Hetero	Reported (Miyagawa et al., 2013; Oh et al., 2019)
	M4	*USH2A*	13	Missense	c.2802T>G	C934W	Hetero	Reported (Miyagawa et al. 2013; Neveling et al., 2012)
5	M9	*USH2A*	63	Missense	c.13465G>A	G4489S	Hetero	Novel
	M10	*USH2A*	2	Insertion	c.99_100insT	R34Sfs	Hetero	Reported (McCulloch et al., 2015)

Abbreviation: Hetero, heterozygous.

#### 3.3.1 Location of the novel mutations in *USH2A*

The p.C931Y mutant is located within the laminin EGF‐like domain (Lam EGF) of USH2A, and the p.G4489S and p.M4853V mutants are located within the fibronectin type III domain (FN3). Figure S1 (in the supplemental materials) shows the schematic diagram of the reported mutations along the USH2A protein domains (without showing the novel intronic mutation IVS22+3A>G).

#### 3.3.2 Allele frequency

Allele frequency for the nine detected *USH2A* variants (except for IVS22+3A>G) in the general population ranged from 0% to 0.07825% in total. The allele frequency for the three novel missense variants (p.C931Y, p.G4489S, p.M4853V) was 0.005771%, 0.000000%, and 0.005766%, respectively (Table 3).

**TABLE 3:** Pathogenicity clues of the variant detected in the *USH2A* gene in this study

*USH2A* Mutation	Protein domain	Allele frequency	Conservative	PolyPhen‐2	PROVEAN	Grantham score	SIFT	Patho‐genicity
R34Sfs	Signal peptide	0.000000%	0.244	NA	NA	NA	NA	Pathogenic
C931Y	Lam EGF	0.005771%	0.858	Probably damaging (0.999)	Deleterious	Radical (194)	Damaging (0.000)	Pathogenic
C934W	Lam EGF	0.001649%	0.858	Probably damaging (0.999)	Deleterious	Radical (215)	Damaging (0.000)	Pathogenic
G1861S	FN3	0.000831%	0.638	Probably damaging (1.000)	Deleterious	Radical (159)	Damaging (0.000)	Pathogenic
R2853G	FN3	0.000000%	0.701	Probably damaging (0.999)	Deleterious	Moderately radical (125)	Damaging (0.015)	Pathogenic
G4489S	FN3	0.000000%	0.685	Probably damaging (1.000)	Deleterious	Moderately conservative (56)	Tolerated (0.069)	Likely pathogenic
Y4673H	FN3	0.000000%	0.591	Possibly damaging (0.754)	Deleterious	Moderately conservative (83)	Tolerated (0.146)	Likely pathogenic
M4853V	FN3	0.005766%	0.134	Benign (0.010)	Neutral	Conservative (21)	Tolerated (0.424)	Likely benign
R5143C	TM	0.07825%	0.646	Benign (0.031)	Deleterious	Radical (180)	Damaging (0.029)	Likely pathogenic
IVS22+3A>G	Intron	NA	NA	NA	NA	NA	NA	Pathogenic

**PolyPhen‐2** (polymorphism phenotyping v2): “Probably damaging” (0.909–1, it is believed most likely to affect protein function or structure), “Possibly damaging” (0.447–0.908, it is believed to affect protein function or structure), “Benign” (0–0.446, most likely lacking any phenotypic effect).

**PROVEAN** (protein variation effect analyzer): Variants with a score equal to or below −2.5 are considered “deleterious”; Variants with a score above −2.5 are considered “neutral.”

**Grantham scores**, which categorize codon replacements into classes of increasing chemical dissimilarity, were designated conservative (0–50), moderately conservative (51–100), moderately radical (101–150), or radical (≥151) according to the classification proposed by Li et al (Jaijo et al., 2010; Jiang et al., 2015).

**SIFT** (Sort Intolerant From Tolerant): Ranges from 0 to 1. The amino acid substitution is predicted damaging if the score is <= 0.05 and tolerated if the score is >0.05.

Abbreviations: FN3, fibronectin type III; Lam EGF, laminin EGF‐like domain; NA, not applicable; TM, transmembrane region.

#### 3.3.3 Conservative prediction

**FIGURE 3:**
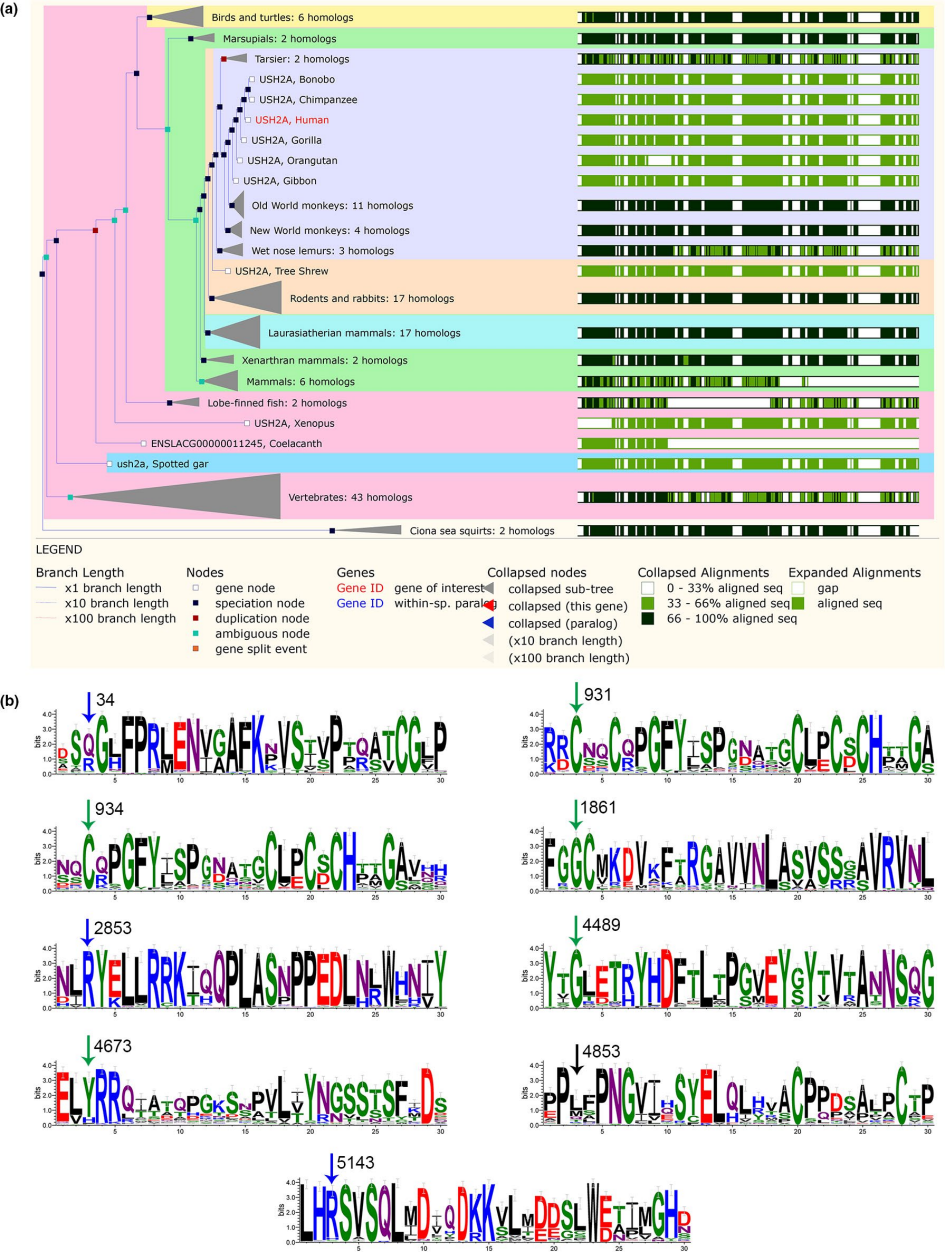
The phylogenetic tree of the *USH2A* gene and the conservative prediction of each mutation site. (a) The phylogenetic tree shows the sequence of multiple alignments of the *USH2A* homologous family. (b) The seqlogo map of amino acid sequences around the mutation point and their conservativeness. The third amino acid in the diagram belongs to the amino acid corresponding to each mutation point

A total of 127 homologous genes of *USH2A* taken from 127 species were used to construct the phylogenetic tree (Figure 3a). The human *USH2A* homolog located in the same sub‐clade together with that in the primate Bonobo and Chimpanzee, suggesting these *USH2A* genes share the most evolutionary similarity with each other. The WebLogo software draws the seqlogo diagram of the sequence around the amino acids corresponding to each mutation point. As shown in Figure 3b, the third amino acid in the diagram indicates the amino acid corresponding to each mutation point. The larger the letter, the more conservative the amino acid at this site is. The conservative calculation of amino acids corresponding to each mutation site in the phylogenetic tree can be found in Table 3. Among the nine available mutation sites on *USH2A*, seven had conservative values greater than 0.5, while the amino acids at the 34 and 4853 sites had conservative values less than 0.5.

#### 3.3.4 Pathogenicity prediction

The three novel missense variants (p.C931Y, p.M4853V, and p.G4489S) could not be found in the 1000 Genomes Database. They were predicted to be deleterious (p.G4489S and p.C931Y), and neutral (p.M4853V) by PROVEAN; to be probably damaging (p.G4489S and p.C931Y) and benign (p.M4853V) by PolyPhen‐II; to affect protein function (p.C931Y) and to be tolerated (p.G4489S and p.M4853V) by SIFT; and to be radical (p.C931Y), moderately conservative (p.G4489S) and conservative (p.M4853V) by Grantham scores. The bioinformatics analyses of the *USH2A* variants are summarized in Table 3.

Additionally, IVS22+3A>G is a novel splicing variant that could be considered pathogenic. The prediction and analysis of the secondary structure of DNA around the intron mutation IVS22+3A>G showed that the mutation position formed a hairpin structure (Figure S2 in the supplemental materials, circled in red). Hairpin loop structures are an important structural motif in nucleic acids that have been shown to play important roles in many biological processes (Kannan & Zacharias, 2007).

#### 3.3.5 Protein structure modeling and analysis

**FIGURE 4:**
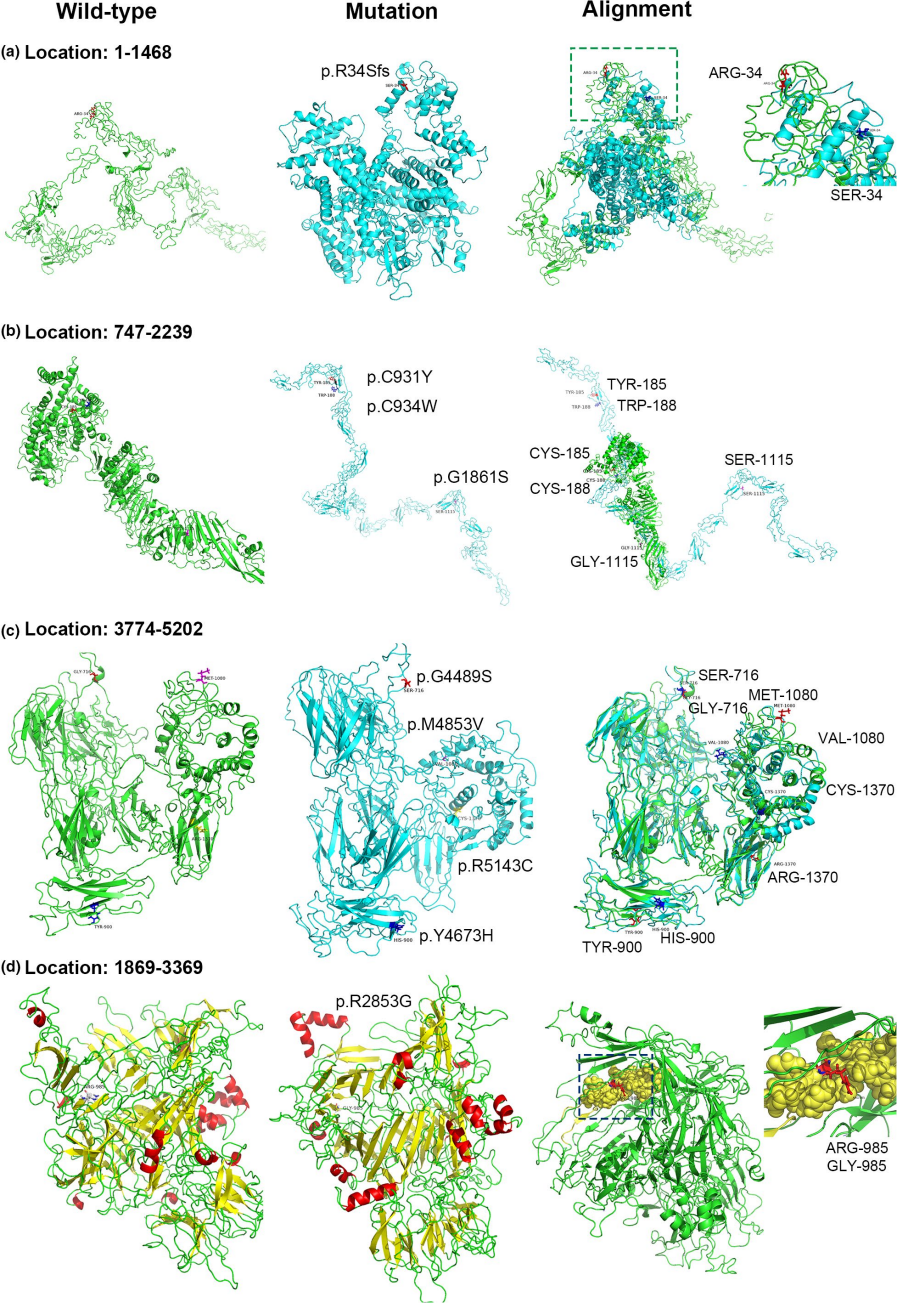
Three‐dimensional structural modeling of wild‐type and mutant USH2A protein in four regions. (a) Region 1 (1‐1468 amino acid): p.R34Sfs (c.99_100insT); (b) Region 2 (747‐2239): p.C931Y (c.2792G>A), p.C934 W (c.2802T>G), p.G1861S (c.5581G>A); (c) Region 3 (3774‐5202): p.G4489S (c.13465G>A), p.Y4673H (c.14017T>C), p.M4853V (c.14557A>G), p.R5143C (c.15427C>T); (d) Region 4 (1869‐3369): p.R2853G (IVS42‐2A>G)

Three‐dimensional structural modeling of the wild‐type and mutant USH2A proteins were cut in four regions according to the principle of covering the mutation point to the greatest extent (Table S1 in the supplemental materials). Region 1 (amino acids 1‐1468) covers code‐shifting mutations caused by insertions at bases 99‐100: p.R34Sfs (c.99_100insT); region 2 (amino acids 747‐2239) covers three missense mutations, including p.C931Y (c.2792G>A), p.C934W (c.2802T>G), and p.G1861S (c.5581G>A); region 3 (amino acids 3774‐5202) covers four missense mutations, including p.G4489S (c.13465G>A), p.Y4673H (c.14017T>C), p.M4853V (c.14557A>G), and p.R5143C (c.15427C>T); and region 4 (amino acids 1869‐3369) covers the splicing mutation in exon 42: IVS22+3A>G. Figure 4 shows the three‐dimensional structural modeling of the wild‐type and mutant USH2A proteins and their alignments in these four regions.

#### 3.3.6 Protein function prediction in mutation point

The amino acid positions 922 and 931 as well as 934 and 948 were found to form disulfide bonds (Figure S3 in the supplemental materials). The predicted results showed that the reliability of disulfide bond formation is 1. In our study, the amino acids at positions 931 and 934 of the USH2A protein were the sites containing the gene mutations, and it can be assumed that the mutations at amino acids 931 and 934 will lead to disulfide bond breaking and thereby affect the structure of the USH2A protein.

## 4 DISCUSSION

The *USH2A* gene is located on chromosome 1q41 and encodes the usherin protein, which is expressed in the supportive tissue in the inner ear and retina. The usherin protein plays a critical role in the development of cochlear hair cells and the long‐term maintenance of photoreceptors (van Wijk et al., 2004; Weston et al., 2000). In addition, it is functionally connected with the other proteins in the interactome of the retina (Sodi, Mariottini, & Passerini, 2014).

However, it remains unknown why some mutations in *USH2A* result in Usher syndrome type IIa, while others develop nonsyndromic RP (Pierrache et al., 2016). A growing number of studies have shown that the total loss of function of the USH2A protein predisposes patients to Usher syndrome type IIa but that remnant protein function can lead to RP with or without hearing loss, indicating that mutations in the *USH2A* gene are relatively less harmful in nonsyndromic RP than in Usher syndrome type IIa. A multicenter research performed in the Netherlands and Belgium noted that truncating mutations are restricted to the Usher syndrome phenotype (Pierrache et al., 2016).

Nonsyndromic *USH2A* retinopathy is a primary cause of adult‐onset recessive retinal degeneration (Sandberg et al., 2008). In our study of nonsyndromic RP, we also found that the severity of the disease (an earlier visual decline) is related to the severity of the gene mutation as follows: insertion > splicing > missense mutation (PROVEAN prediction: deleterious > neutral). The proband of pedigree 5 had an insertion mutation in *USH2A* gene and had disease‐related visual impairment at the age of 12; Probands of pedigrees 2 and 3 had splicing mutations in *USH2A* gene and had visual decline around the age of 35; while probands of pedigrees 1 and 4 had missense mutations and had visual impairment around the age of 45 (Tables 1 and 2). The larger sample size will tell more about these correlations. In addition, our study shows that the severity of visual impairment observed in nonsyndromic RP patients with *USH2A* mutations is related to the progression of the disease (Table 1). Most *USH2A*‐associated RP patients developed severe visual impairment (best‐corrected visual acuity (BCVA) ≤0.05, legally blind) around the age of 50 years old, consistent with previous reports (Pierrache et al., 2016; Sandberg et al., 2008).

*USH2A*‐associated RP patients show variable clinical characteristics (different ages of onset, disease progression, and fundus appearance), and it was, therefore, difficult to establish reliable genotype‐phenotype correlations (Sodi et al., 2014). In addition, most patients are compound heterozygotes as a result of carrying different mutations on the maternal and paternal allele, making it even more difficult to predict the effect of each of these mutations on the phenotype and complicating attempts to evaluate a possible allelic hierarchy (Lenassi et al., 2015).

Ultra‐wide imaging may provide more information about the contrast between the posterior pole and mid‐periphery of the retina, so it can better assist in clinical diagnosis. Increased posterior pole autofluorescence occurs with increased accumulation of lipofuscin in the RPE or loss of rod outer segments (Lorenz et al., 2004; Trichonas, Traboulsi, & Ehlers, 2017). And the enhancement of parafoveal autofluorescence in FAF corresponds to the thinning of the outer retina in optical coherence tomography (OCT). Therefore, hyperautofluorescence parafoveal ring with macular sparing may be an imaging biomarker for genotype association in retinal dystrophies and RP. Different fundus manifestations (phenotypes) may be due to the different stages of RP, rather than to different genotypes.

Besides, angiographically silent cystoid macular edema presented in pedigree 1 (Figure 2 and Figure S4) has been previously reported in other cases (Le Quesne Stabej et al., 2012). Cystoid macular edema secondary to retinal degenerative diseases can easily lead to misdiagnosis, and this type of macular edema has a poor response to anti‐VEGF therapy. Therefore, it is helpful to diagnose RP by grasping distinctive patterns, combining with clinical symptoms, electroretinogram (ERG), and visual field changes.

As for *USH2A* gene mutation and its effect on protein function, Caroline C.W. Klaver et al studied the *USH2A* genotypes and found that less than 19% of mutations were homozygous and 69% were private, which suggests that the heterogeneity is very large and those novel mutations may occur frequently (Pierrache et al., 2016). The high prevalence of de novo variants and the poor rate of variants common in other ethnic groups indicate a unique mutational spectrum of the *USH2A* gene in Chinese patients with nonsyndromic RP (Sodi et al., 2014).

In our study, interestingly, the proband (II:2) and her affected older brother (II:3) in pedigree 1 were found to be heterozygous carriers of M1 and M2 mutations (on the same chromatid) in the *USH2A* gene, associated with retinitis pigmentosa without hearing loss. Although there is high prevalence of carriers in inherited retinal dystrophies mutations (González‐Del Pozo et al., 2018), such a carrier state is hard to cause retinitis pigmentosa independently. Liquori A et al. have pointed out that deep intronic mutations in *USH2A* are underestimated. And unfortunately, analyzing *USH2A* transcripts is challenging and for 1.8%–19% of Usher syndrome type II individuals who carry a single *USH2A* recessive mutation, a second mutation is yet to be identified (Liquori et al., 2016). The probability of an additional retinal dystrophy gene mutation conferring a compound heterozygote state may also elucidate the presence of extensive retinal degeneration in this case (Rivolta, Sweklo, Berson, & Dryja, 2000). Other possible mutations associated with retinal dystrophy found in the comprehensive genetic testing of the proband in Pedigree 1 are referred to supplemental materials (Table S2 in the supplemental materials). However, whether these mutations are deleterious requires further family members Sanger sequencing and functional experiments to verify.

Other studies have also illustrated this possibility (Cremers et al., 2007; Jaijo et al., 2010; Le Quesne Stabej et al., 2012; Sodi et al., 2014; Västinsalo et al., 2013; Vozzi, Aaspõllu, & Athanasakis, 2011). A study based in Italy found that one patient received the *USH2A* variant from the unaffected father and the MYO7A variant from the unaffected mother, which was heterozygous for mutations in both USH genes (Sodi et al., 2014). Similar findings in other studies support the hypothesis of a possible digenic/oligogenic inheritance of the syndrome/nonsyndromic RP or the possible joint impact of variants of different genes on the clinical phenotype of the patient. Unrecognized mutations could present in the promoter, regulatory regions, and deep intronic areas, usually not tested during conventional mutation screening (Sodi et al., 2014).

Our study has several limitations. It is a retrospective study that does not provide enough information on time‐dependent longitudinal changes. In addition, the number of patients with mutations in the same gene places restrictions on quantitative and statistical analysis, as well as further delineation of genotype‐phenotype correlation. Moreover, although *in silico* prediction, protein modeling and disulfide bond function prediction of de novo mutations have been carried out, there is still a lack of further cellular and animal functional validation partly due to the difficulty of large protein synthesis and point mutation.

## 5 CONCLUSIONS

In conclusion, this study identified four novel mutations of the *USH2A* gene using a whole‐exome sequencing approach, which further confirms that USH2A protein plays a pivotal role in the maintenance of photoreceptors and expands the spectrum of *USH2A* mutations that are associated with nonsyndromic RP in Chinese patients. Furthermore, we are the first to construct the tertiary structure of wild‐type and mutant USH2A proteins by three‐dimensional protein structural modeling and analysis, which makes the changes in protein structure caused by different mutations observed directly. Nevertheless, for some mutations with uncertained pathogenicity, further functional experiments are needed to validate.

Through the evaluation and analysis of ultra‐wide‐field fundus images, we found that nonsyndromic RP caused by *USH2A* mutation has the same distinctive pattern of hyperautofluorescence parafoveal ring with macular sparing. Meanwhile, we clarified that different fundus appearances are only the characteristics of different stages of RP, not different phenotypes caused by different genotypes. Better knowledge of the molecular genetic background underlying nonsyndromic RP in specific populations may contribute to more efficient diagnostic strategies and future therapeutic approaches.

## 6 ETHICS APPROVAL

This study was approved by the medical ethics committee of Shanghai General Hospital, Shanghai Jiao Tong University (No. 2019KY044), and conducted in accordance with the Declaration of Helsinki. The data presented in this study were procured through retrospective chart review and details are not identifiable to any individual patient.

## CONFLICT OF INTEREST

The authors declare no conflict of interest. The funding organizations had no role in the design of the study; in the collection, analyses, or interpretation of data; in the writing of the manuscript; and in the decision to publish the results.

## AUTHOR CONTRIBUTIONS

Chong Chen and Qiao Sun contributed equally to this study and are listed as co‐first authors. Suqin Yu and Xun Xu had full access to all the data in the study and take responsibility for the integrity of the data and the accuracy of the data analysis. Conception and design: Suqin Yu, Chong Chen. Data collection: Chong Chen, Qiao Sun, Tianwei Qian, Dawei Luo, Suqin Yu. Analysis and interpretation: Chong Chen, Qiao Sun, Mingmin Gu, Kun Liu, Xun Xu, Suqin Yu. Drafting the manuscript: Chong Chen, Suqin Yu. Critical revision of the manuscript: Suqin Yu, Xun Xu, Mingmin Gu, Qiao Sun, Kun Liu. Supervision: Xun Xu, Suqin Yu.

## Supporting information

Supplementary Material
