# Supplementary material for: Multimodal imaging and genetic characteristics of Chinese patients with USH2A‐associated nonsyndromic retinitis pigmentosa
Source: Mol Genet Genomic Med. 2020 Sep 6;8(11):e1479. doi: 10.1002/mgg3.1479 (PMC7667352; doi:10.1002/mgg3.1479)
Supplement: Supplementary file 1 — Supplementary Material [file MGG3-8-e1479-s001.docx]

**SUPPLEMENTAL MATERIALS**

**Clinical and genetic details of each pedigree:**

Pedigree 1

A 47-year-old woman (II:2, proband) with no significant medical or family history presented to our department for blurred vision for seven months. She reported no photopsia, no nyctalopia, and no peripheral vision problems. Her BCVA was 0.25 in the right eye and 0.3 in the left eye. Ultrawide-field color photography showed no obvious abnormality. Ultrawide-field FAF showed punctate pigment scattered around the optic disc and subtle peripheral stippling of the RPE. SD-OCT showed perifoveal loss of the ellipsoid zone with central preservation and cystoid macular edema (Figure 2, pedigree 1). Ultrawide-field FFA showed fluorescein leakage from retinal vessels and a hyper-fluorescent ring in the posterior pole with foveal sparing, although it failed to demonstrate cystoid macular edema (Figure 3 in the supplemental materials). Findings were symmetric in both eyes. Her hematologic tests were negative. She was diagnosed with uveitis and treated with oral hormone therapy and biocular anti-VEGF intravitreal injection once before she presented to our department. We gave her anti-VEGF treatment one additional time combined with methazolamide tablets orally for 2 weeks. One month later, her vision acuity had increased to 0.5 binoculus with slightly alleviated macular edema. However, the macular edema was not fully recovered and continues to persist (after 50 months follow-up). Genetic analysis detected a novel variation at c.2792G>A (E13) [p.C931Y] and a missense mutation at c.14017T>C (E64) [p.Y4673H] of the *USH2A* gene. These mutations were further confirmed by Sanger sequencing. Additional testing demonstrated that p.C931Y (M2) and p.Y4673H (M1) replacements were also presented in the I:1, II:3, III:1 and III:2 individuals in her family (Figure 1, pedigree 1), and of these individuals, II:3 (her big brother) was affected, whereas I:1 (her father) was unable to submit to ophthalmic exams due to his age and distance, and III:1 and III:2 (her children) have not yet shown symptoms such as nyctalopia, and they therefore refused to be further examined. Whether their symptoms and signs will be late-onset, as was found in their mother (onset at 46 years old), remains to be determined. These genetic data suggest that the phenotype in this family may follow an autosomal dominant inheritance pattern or that an additional retinal dystrophy gene mutation that confers a compound heterozygote state may reveal the presence of extensive retinal degeneration in this case.

Pedigree 2

A 37-year-old woman (II:1, proband) with blurred vision and peripheral vision problems in both eyes presented to our department. She reported no photopsia or nyctalopia. Her BCVA was 1.0 in the right eye and 0.9 in the left eye. Ultrawide-field color photography showed localized retinal atrophy below the optic disc and extensive peripheral retinal atrophy as well as attenuated retinal vessels. Ultrawide-field FAF showed bone spicule-shaped pigment deposits in the mid periphery of the retina and a hyper-autofluorescence ring in the perimacular area, while the macula was preserved. The retinal vessels were attenuated. Macular SD-OCT showed perimacular outer retinal atrophy (Figure 2, pedigree 2). Findings were symmetric in both eyes. Genetic analysis detected an IVS42-2A>G splice site mutation and a missense variation at c.2802T>G (E13) [p.C934W] of the *USH2A* gene (heterozygous mutation). The mutation was further confirmed by Sanger sequencing. Parental testing confirmed the biallelic nature of these variants. Her father (I:1) had an IVS42-2A>G splice site mutation (M3), and her mother (II:2) had a missense variation at c.2802T>G (E13) [p.C934W (M4)] of the *USH2A* gene (Figure 1, pedigree 2).

Pedigree 3

A 60-year-old man (II:4, proband) with blurred vision for ten years in both eyes presented to our hospital for an ophthalmic examination. His BCVA was 0.03 in the right eye and 0.05 in the left eye. Ultrawide-field color photography showed bone spicule-shaped pigment deposits presented in the mid periphery of the retina along with retinal atrophy, while the macula was preserved. The retinal vessels were attenuated. Ultrawide-field FAF showed a mottled appearance of the RPE caused by bone spicule formation in the mid periphery of the retina without macular involvement. The retinal vessels were also attenuated. Macular SD-OCT showed perifoveal outer retinal atrophy (Figure 2, pedigree 3). Findings were symmetric in both eyes. Genetic analysis detected a novel IVS22+3A>G splice site mutation and a novel missense variation at c.14557A>G (E66) [p.M4853V] of the *USH2A* gene (heterozygous mutation). Both of his parents passed away.

Pedigree 4

A 31-year-old man (II:1, proband) with blurred and decreased vision presented to our hospital. His BCVA was 1.0 in the right eye and 0.9 in the left eye. Ultrawide-field color photography showed bone spicule-shaped pigmented deposits presented in the mid periphery of the retina, and retinal atrophy with macula sparing, although there was scattered depigmentation in the nasal retina. There was dense chorioretinal atrophy extending from the optic disc, and the optic nerve have a waxy pallor. Ultrawide-field FAF showed a lobular RPE defect due to bone spicule formation in the mid periphery of the retina without macular involvement. The perifoveal area showed a hyper-autofluroscent ring. Besides, there were alterations in the peripheral retinal vasculature. Macular SD-OCT showed perifoveal outer retinal atrophy as well as intraretinal cysts (Figure 2, pedigree 4). Findings were symmetric in both eyes. Genetic analysis detected a c.15427C>T (E71) [p.R5143C] variant, a c.5581G>A (E28) [G1861S] variant, and a variant at c.2802T>G (E13) [p.C934W] of the *USH2A* gene. This last variant was identical to that found in case 2. Additional Sanger sequencing demonstrated that his father (I:1) had the p.R5143C (M7) and p.G1861S (M8) variants, while his mother (I:2) possessed the p.C934W (M4) mutation (Figure 1, pedigree 4).

Pedigree 5

A 54-year-old woman (II:1, proband) with blurred vision presented to our department. Her BCVA was 0.03 in the right eye and 0.01 in the left eye. Ultrawide-field color photography showed circumferential lacy-like spicules of pigment epithelial hyperplasia extending into the mid and far peripheral fundus along with retinal atrophy, while the macula was preserved with a perifoveal ring of depigmentation. There was also dense chorioretinal atrophy extending from the optic disc and generalized arteriolar narrowing, and the optic disc had a waxy appearance. Ultrawide-field FAF showed patchy RPE defects confluent into a zonal area of atrophy in the mid periphery of the retina with macular sparing. The perifoveal area showed a hyperautofluroscent ring with retinal vessels that were almost invisible. Macular SD-OCT showed perifoveal outer retinal atrophy (Figure 2, pedigree 5). Findings were symmetric in both eyes. Genetic analysis detected a novel c.13465G>A (E63) [p.G4489S] variant and an insertion at c.99_100insT (E2) [p.R34Sfs] of the *USH2A* gene. Both of her parents passed away.

**T**
PLEMENTARY MATERIALS4853V)pes. PXXXXXXXXXXXXXXXXXXXXXXXXXXXXXXXXXXXXXXXXXXXXXXXXXXXXXXXXXXXXXXXXXXXXXXXXXXXXXXXXXXXXXXXXXXXXXXXXXXXXXXXXXXXXXXXXXXXXXXXXXXXXXXXXXXXXXXX**region**XXXXXXXXXXXXXXXXXXXXXXXXXXXXXXXXXXXXXXXXXXXXXXXXXXXXXXXXXXXXXXXXXXXXXXXXXXXXXXXXXXXXXXXXXXXXXXXXXXXXXXXXXXXXXXXXXXXXXXXXXXXXXXXXXXXXXXX**ABLES**

**Table S1.** The structure of the USH2A protein was cut into 4 regions for three-dimensional modeling.

| Region | Location | Covering the cDNA | Exons | Alteration of Amino Acids | Base position |
| --- | --- | --- | --- | --- | --- |
| 1 | 1-1468 | c.99_100insT | 2 | R34Sfs | 99 |
| 2 | 747-2239 | c.2792G>A | 13 | C931Y | 2792 |
|  | 747-2239 | c.2802T>G | 13 | C934W | 2802 |
|  | 747-2239 | c.5581G>A | 28 | G1861S | 5581 |
| 3 | 3774-5202 | c.13465G>A | 63 | G4489S | 13465 |
|  | 3774-5202 | c.14017T>C | 64 | Y4673H | 14017 |
|  | 3774-5202 | c.14557A>G | 66 | M4853V | 14557 |
|  | 3774-5202 | c.15427C>T | 71 | R5143C | 15427 |
| 4 | 1869-3369 | IVS42-2A>G | 42 | R2853G | 8557 |

**Table S2.** Other gene mutations with unknown clinical significance detected by whole exon sequencing of the proband in Pedigree 1

| Gene | Exon | cDNA | Amino acid | Provean | Associated disease phenotype (Inheritance) |  |
| --- | --- | --- | --- | --- | --- | --- |
| IMPG2 | 1 | c.-185A>C | Non-coding region | UTR mutation may affect protein translation | RP type 56 (AR) |  |
| CTSD | 6 | c.756_c.757insC | p.K253Qfs*39 | Frameshift mutation | Neuronal ceroid lipofuscinosis type 10 | |
| CDH23 | 5 | c.595G>A | p.E199K | Possibly damaging | Usher Syndrome type ID (AR) | |
| PCDH15 | 12 | c.1210A>C | p.S404R | Possibly damaging | Usher Syndrome type ID /IF (AR) | |
| PDE6B | 1 | c.145G>T | p.D49Y | Possibly damaging | RP type 40 (AR) | |
| CNGB1 | 18 | c.1631C>T | p.P544L | Possibly damaging | RP type 45 (AR) | |
| EYS | 32 | c.6562A>G | p.I2188V | Neutral | RP type 25 (AR) | |
| DFNB31 | 9 | c.1999G>A | p.A667T | Neutral | Usher Syndrome type IID | |
| NPHP4 | 3 | c.203G>A | p.R68Q | Neutral | Senior-Loken syndrome (Kidney-retinal dystrophy) type 4 | |

**FIGURES**


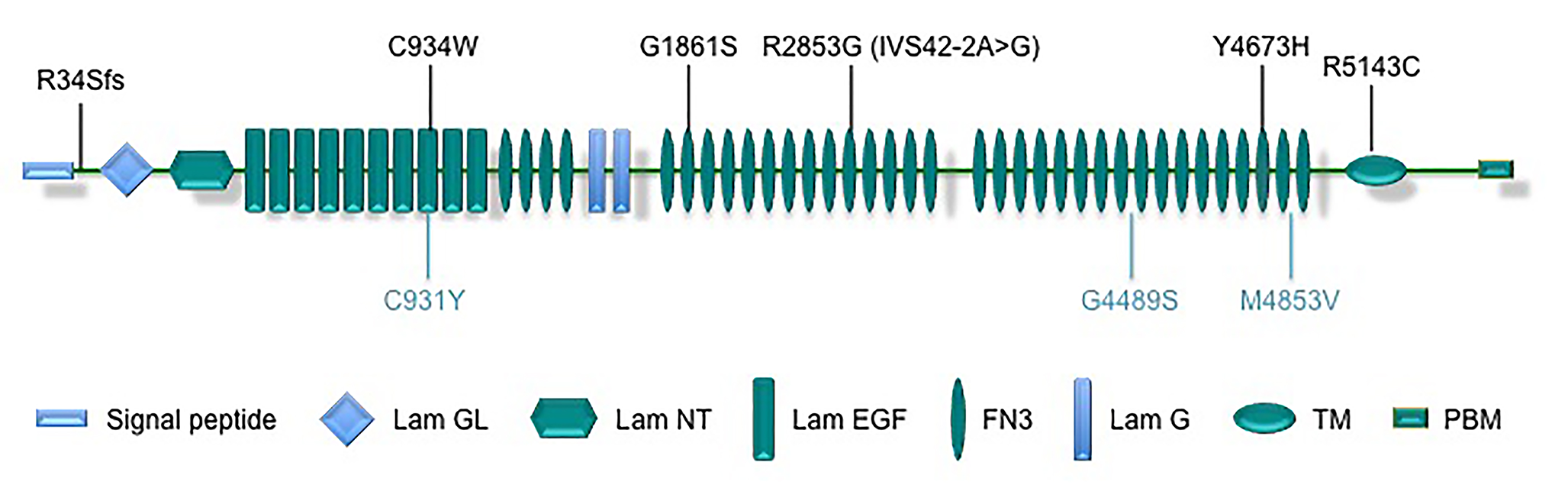


**Figure S1.** Schematic diagram of the mutations in this study along the USH2A protein domains. Six disease-causing mutations in *USH2A* were from previous studies (black characters), and four novel mutations were identified in this study (light sea green characters, p.C931Y, p.G4489S and p.M4853V, without showing intronic mutation: IVS22+3A>G). Lam GL: Laminin G-like domain; Lam NT: Laminin N-terminal; Lam EGF: Laminin EGF-like domain; FN3: Fibronectin type III; Lam G: Laminin G domains; TM: Transmembrane region; PBM: PDZ-binding motif.


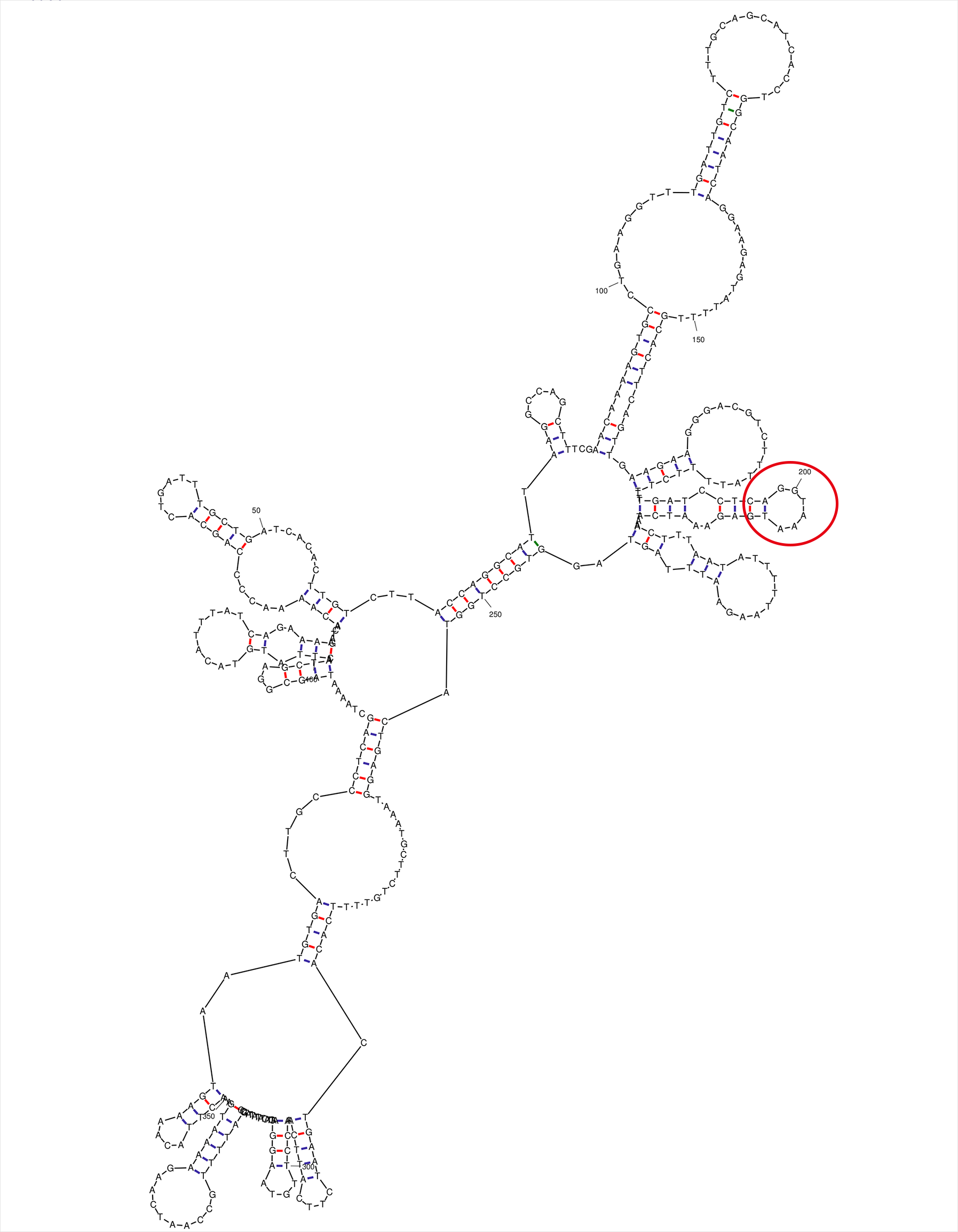


**Figure S2.** Prediction and analysis of secondary structure of DNA around intron mutation IVS22+3A>G. The mutation position is circled in red and formed a hairpin structure.

**
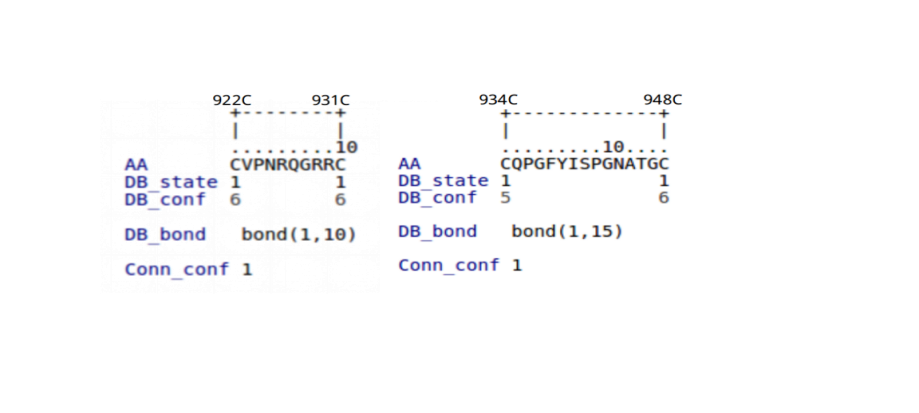
**

**Figure S3**. Diagram of disulfide bond results of USH2A protein predicted by DISULFIND software.


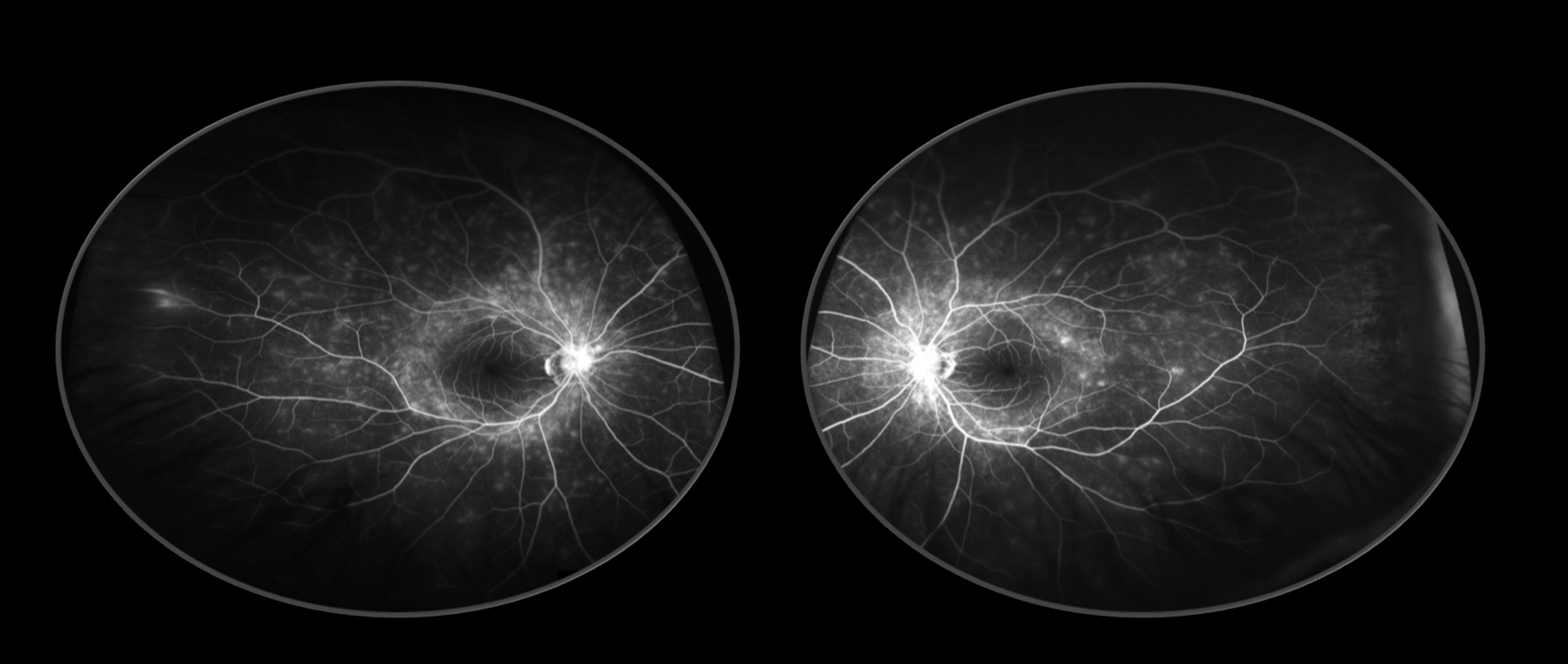


**Figure S4**. Ultrawide-field fluorescein angiography of prophet in pedigree 1. Findings were symmetric on both eyes.
